# Association between oxidative balance score and sarcopenia in older adults

**DOI:** 10.1038/s41598-024-56103-4

**Published:** 2024-03-04

**Authors:** Marzieh Mahmoodi, Zainab Shateri, Seyed Alireza Nazari, Mehran Nouri, Nasrin Nasimi, Zahra Sohrabi, Mohammad Hossein Dabbaghmanesh

**Affiliations:** 1 Department of Clinical Nutrition, School of Nutrition and Food Sciences, Shiraz University of Medical Sciences, Shiraz, Iran; 2 Student Research Committee, Shiraz University of Medical Sciences, Shiraz, Iran; 3 Department of Nutrition and Biochemistry, School of Medicine, Ilam University of Medical Sciences, Ilam, Iran; 4 School of Advanced Technologies in Medicine, Tehran University of Medical Sciences, Tehran, Iran; 5 Mobility Impairment Research Center, Health Research Institute, Babol University of Medical Sciences, Babol, Iran; 6 Department of Community Nutrition, School of Nutrition and Food Sciences, Shiraz University of Medical Sciences, Shiraz, Iran; 7 Nutrition Research Center, School of Nutrition and Food Sciences, Shiraz University of Medical Sciences, Shiraz, Iran; 8 Endocrinology and Metabolism Research Center, Shiraz University of Medical Sciences, Shiraz, Iran

**Keywords:** Oxidative balance score, Sarcopenia, Older adults, Elderly, Iranian, Biomarkers, Diseases, Health care, Medical research

## Abstract

Sarcopenia is a progressive skeletal muscle disease in which oxidative stress has been proposed as one of the primary markers. The oxidative balance score (OBS) represents the oxidative balance of a person's dietary pattern using the merged intake of anti-oxidants and pro-oxidants. Therefore, the present study assessed the association between OBS and sarcopenia in Iranian older adults. In the current study, 80 people with sarcopenia and 80 without it were considered the case and control groups, respectively. All controls were matched by sex with cases. To confirm sarcopenia, skeletal muscle mass index (SMI), handgrip strength (HGS) measurement, and gait speed were used. Also, body composition was measured by bioelectrical impedance analysis (BIA). A valid and reliable food frequency questionnaire (FFQ) was used to assess all participants' dietary intake of pro-oxidants and anti-oxidants. Conditional logistic regression was applied to assess the association between OBS and sarcopenia. In the bivariate model, we observed lower odds of sarcopenia in the second and last tertile of OBS in comparison to the first tertile (T) (T_2_ – odds ratio (OR) = 0.414, 95% confidence interval (CI) : 0.186–0.918 and T_3_ – OR = 0.101, 95% CI: 0.041–0.248). After adjusting for potential confounders, the association was not significant in second and last tertile of OBS in comparision to the first one. The present study's findings demonstrated that overcoming exposure to anti-oxidants over pro-oxidants, as illustrated by a higher OBS, is not related to lower odds of sarcopenia in older adults.

## Introduction

Sarcopenia is a progressive skeletal muscle disease in which muscle function is disrupted due to the loss of muscle mass. Adverse consequences of this disease include decreased performance, frailty, falls, and death^1^. The prevalence of this disease is estimated between 10 and 27% in people over 60 years old^2^.

Sarcopenia is more common in older adults^3^^,^ but muscle mass decreases by age 40^4^. Sarcopenia has a complex pathophysiology and can occur as a result of an increase in apoptotic activity of myofibrils, a reduction in the number of α-motor neurons, hormonal imbalance (a decrease in anabolic hormones), an increase in proinflammatory cytokines, biological changes, changes in mitochondrial function, increased oxidative stress, and factors such as energy deficiency^5,6^. One of the primary markers proposed for sarcopenia is oxidative stress^7^. Oxidative stress is an imbalance between the oxidant and anti-oxidant systems of the body^8^. Oxidative stress can be the main cause of sarcopenia as a result of the excessive production of mitochondrial radicals due to the reduction of anti-oxidant enzymes in muscle cells^9^.

The oxidative balance score (OBS) represents the oxidative balance of a person's dietary pattern using the merged intake of anti-oxidants and pro-oxidants^10^. So far, studies on the role of oxidative stress in diseases such as colorectal adenoma^11^, colorectal cancer^12^, prostate cancer^13^, hypertension^14^, and osteoporosis^15^ have been conducted using this index. However, to our knowledge, no study has examined the association between OBS and sarcopenia. But, some studies have investigated the parameters of oxidative stress in sarcopenia. A cross-sectional study showed that glutathione peroxidase in diabetic patients with sarcopenia was significantly lower than in the control group^16^. Also, xanthine oxidase was significantly higher in people with sarcopenia than in the control group^16^. In contrast, a study revealed no significant difference in the serum levels of thioredoxin-1 (a protein protecting the cell against oxidative stress) in people with sarcopenia compared to non-sarcopenia^17^.

Despite the role of oxidative stress in the pathogenesis of sarcopenia, no studies have been conducted on the association between OBS and the risk of sarcopenia in older adults. Therefore, the present study aimed to assess the association between OBS and sarcopenia in Iranian older adults.

## Methods

### Study population

We conducted a case-control study, a subset of a previous cross-sectional study, on the older adult population referring to healthcare centers in Shiraz, Iran, from August 2017 to February 2018^18^. In summary, in the case group, there were 80 people with sarcopenia confirmed in the previous cross-sectional study, and in the control group, there were 80 people without sarcopenia. All controls were matched by sex with cases. Also, all participants without cognitive problems (including history of Alzheimer's disease and dementia) were included in the present study. However, incompleteness of the food-frequency questionnaire (FFQ) or unwillingness to participate in the study were exclusion criteria. All participants completed the written informed consent before their inclusion in the study. The ethics committee of Shiraz University of Medical Sciences approved the current study, and it was carried out in line with the principles of the Declaration of Helsinki (Code: 27983).

Demographic data about sex, age, education, and smoking status for all participants were collected through a checklist. Also, using a digital scale, participants’ weight was measured according to standard methods. Also, height was measured by a tap meter according to standard methods. Body mass index (BMI) was determined as weight (kg) divided by the square of height (meters). Also, the International Physical Activity Questionnaire (IPAQ) was used for physical activity assessment^19^.

### Sarcopenia diagnosis

A diagnosis of low muscle mass, low muscle strength, and/or low muscle function was necessary to confirm the presence of sarcopenia, according to the Asian Working Group on Sarcopenia (AWGS) guidelines^18,20^. Based on AWGS guidelines, individuals with low skeletal muscle mass and low muscle strength or low physical function were considered to have sarcopenia, and individuals with low skeletal muscle mass and both low muscle strength and low physical function were considered to have severe sarcopenia^18^^,^^20^.

Body composition was determined by bioelectrical impedance analysis (BIA) using an InBody S10 analyzer (BioSpace Co., Ltd., South Korea). Then, the skeletal muscle mass index (SMI) was calculated by dividing appendicular skeletal muscle mass^21^ by the squared height (meters). SMI of less than 7 kg/m^2^ for men and less than 5.7 kg/m^2^ for women was considered the first step to confirm sarcopenia^18,20^. Muscle strength was determined by measuring handgrip strength (HGS) using a hydraulic hand dynamometer (model MSD, Sihan, Korea). The participants squeezed a hand dynamometer in both hands three times with 15-s pauses in a seated position and 90-degree elbow flexion. The maximum value was used for further analyses. Muscle strength of less than 18 kg for women and less than 26 kg for men was considered a low HGS^18,20^. The participants' muscle function was evaluated using the usual Gait Speed (GS) at a distance of four meters. Each participant was asked to walk the distance without any assistance, and then the time was recorded in seconds by a chronometer. A GS of less than 0.8 m/s was determined as a low physical function indicator^18,20^. All measurements were performed in the morning for all participants, and they were asked not to change their usual dietary pattern and daily physical activity and to refrain from vigorous activities the day before the test.

### Dietary assessment and food grouping

A valid and reliable 168-item FFQ with standard and common serving sizes used by Iranians^22^ was administered to all participants by trained interviewers. Participants were asked to report their daily, weekly, monthly, or yearly intake of food or food items during the past year. Then, all food items were changed to grams based on the methods of Ghaffarpour et al.^23^. Finally, energy intake and all nutrients were extracted by Nutritionist IV software^24^.

Also, OBS was calculated using the following: intake of dietary pro-oxidants such as iron, polyunsaturated fatty acids (PUFAs), and saturated fatty acids (SFAs), non-dietary pro-oxidants including smoking and obesity, dietary anti-oxidant intakes such as fibers, folate, vitamin C, vitamin E, beta-cryptoxanthin, lycopene, lutein/zeaxanthin, alpha-carotene, beta-carotene, selenium, and zinc, and non-dietary anti-oxidants such as physical activity ^25–28^. This method was suggested by Goodman et al. ^29^, and at first, the dietary intake of each item was changed to tertile, and the score was based on Table 1. The final score of OBS was between 0.0 and 34.0^30^^,^^31^. The lowest score (0.0) is related to the greater level of exposure to pro-oxidants, and the higher score (34.0) is considered to have a greater level of exposure to anti-oxidants.

**Table 1:** Scoring of OBS components.

OBS components	Score		
Non-dietary antioxidant components			
Physical activity (MET-min/d)	0 = low (1st tertile), 1 = medium (2nd tertile), and 2 = high (last tertile)		
Non-dietary pro-oxidant components			
Obesity	0 = BMI ≥ 30 kg/m^2^ AND WC ≥ 0.88 m in females		
	1 = BMI ≥ 30 kg/m^2^ OR WC ≥ 0.88 m in females		
	2 = BMI < 30 kg/m^2^ AND WC < 0.88 m in females		
Smoking	0 = current, 1 = former, and 2 = never		
Dietary anti-oxidant components			
	1st tertile	2nd tertile	last tertile
Vitamin E (mg)	0 = low	1 = medium	2 = high
Vitamin C (mg)	0 = low	1 = medium	2 = high
Alpha-carotene (µg)	0 = low	1 = medium	2 = high
Beta-carotene (µg)	0 = low	1 = medium	2 = high
Beta-cryptoxanthin	0 = low	1 = medium	2 = high
Lutein (µg)	0 = low	1 = medium	2 = high
Lycopene (µg)	0 = low	1 = medium	2 = high
Vitamin B_9_ (µg)	0 = low	1 = medium	2 = high
Zinc (mg)	0 = low	1 = medium	2 = high
Selenium (µg)	0 = low	1 = medium	2 = high
Fiber (g)	0 = low	1 = medium	2 = high
Dietary pro-oxidant components			
SFA (g)	2 = low	1 = medium	0 = high
PUFA (g)	2 = low	1 = medium	0 = high
Iron (mg)	2 = low	1 = medium	0 = high

*OBS* oxidative balance score, *MET* metabolic equivalent of task, *BMI* body mass index, *WC* waist circumference, *SFA*, saturated fatty acid, *PUFA* polyunsaturated fatty acid.

### Statistical analysis

SPSS (version 24) was used for statistical analysis in the present study. A two-sided p-value ˂ 0.05 was considered as a significance level. The Kolmogorov–Smirnov test was used to evaluate the normality of the data. Continuous parameters were reported as mean ± standard deviation (SD) or median (interquartile range (IQR)), and categorical parameters were reported as frequency or percentage. Paired sample T-test or Wilcoxon U test and McNemar test were used to analyze the continuous and categorical variables of the study population between the case and control groups, respectively. Also, analysis of covariance (ANCOVA) t-test was used to adjust the role of age. The consumption of macronutrient intake based on OBS tertile was assessed by the Kruskal–Wallis U-test. Also, conditional logistic regression models were used to evaluate the association between OBS and sarcopenia in bivariate and multivariable models. In addition, variables with p-value < 0.25 were entered in the multivariable-adjusted model and finally, age, energy, and protein intake were included in the multivariable model.

### Ethics approval and consent to participate

This study was approved by the medical research and ethics committee of Shiraz University of Medical Sciences, and the informed consents were completed by all participants. Also, we confirmed all the methods included in this study were in accordance with the Declaration of Helsinki.

## Results

According to the basic features of the study population in Table 2, there was a significant difference in the median age between the case and control groups (70.0 years in the case and 68.0 in the control group) (P = 0.001). Also, weight, height, BMI, muscle strength, GS, and SMI significantly differed between the case and control groups (P˂0.001 for all). Furthermore, energy intake, OBS, and all components (SFA, PUFA, iron, fiber, vitamin E, folate, vitamin C, alpha-carotene, beta-carotene, beta-cryptoxanthin, lutein, lycopene, zinc, and selenium) were significantly different between both groups and all of them were higher in the control group (P˂0.001 for all, expect selenium).

**Table 2:** The basic characteristics of the study population.

Variables	Case (n = 80)	Control (n = 80)	P-value	P-value*
Age (year)^a^	70.0 (8.0)	68.0 (6.0)	**0.001**	**–**
Sex, %^a^			1.000	–
Male	55.0	55.0		
Female	45.0	45.0		
Education, %^a^			0.719	−
Under diploma	65.0	61.3		
Diploma and higher	35.0	38.8		
Income (Rials) per month, %^a^			0.127	−
Less than 3 million	38.8	36.3		
3–6 million	47.5	37.5		
More than 6 million	13.7	26.2		
Smoking, %^a^			0.718	–
Yes	23.7	27.5		
No	76.3	72.5		
Weight (kg)^b^	59.6 ± 9.2	78.1 ± 9.1	**˂0.001**	**˂0.001**
Height (cm)^b^	157.2 ± 9.8	163.6 ± 8.9	**˂0.001**	**˂0.001**
BMI (kg/m^2^)^b^	24.5 ± 4.1	29.2 ± 3.8	**˂0.001**	**˂0.001**
Muscle strength (kg)^a^	16.0 (10.7)	50.8 (24.2)	**˂0.001**	**˂0.001**
Skeletal muscle index (kg/m^2^)^a^	6.1 (1.4)	7.9 (0.9)	**˂0.001**	**˂0.001**
Gait speed (m/second)^b^	0.70 ± 0.10	1.00 ± 0.95	**˂0.001**	**0.014**
Physical activity (MET-min/week)^a^	429.0 (1020.7)	462.0 (1386.0)	0.315	0.097
Total OBS^1^	15.0 (8.7)	21.0 (7.7)	**˂0.001**	**˂0.001**
Energy (kcal/day)^b^	1329.3 ± 472.9	1861.9 ± 450.4	**˂0.001**	**˂0.001**
SFA (g/day)^a^	10.1 (8.3)	16.3 (4.9)	**˂0.001**	**˂0.001**
PUFA (g/day)^a^	8.7 (4.0)	10.4 (3.9)	**˂0.001**	**0.001**
Iron (mg/day)^b^	8.8 ± 3.2	12.9 ± 3.9	**˂0.001**	**˂0.001**
Fiber (g/day)^b^	26.1 ± 12.5	36.1 ± 11.2	**˂0.001**	**˂0.001**
Vitamin E (mg/day)^b^	10.8 ± 4.1	13.9 ± 3.5	**˂0.001**	**˂0.001**
Folate (µg/day)^b^	347.8 ± 138.1	558.8 ± 163.4	**˂0.001**	**˂0.001**
Vitamin C (mg/day)^a^	122.5 (213.5)	251.5 (231.8)	**˂0.001**	**˂0.001**
Alpha-carotene (µg/day)^a^	180.4 (289.2)	587.7 (626.6)	**˂0.001**	**˂0.001**
Beta-carotene (µg/day)^a^	1482.3 (3334.3)	6007.3 (7929.2)	**˂0.001**	**˂0.001**
Beta-cryptoxantine (µg/day)^a^	297.1 (587.2)	636.9 (543.4)	**˂0.001**	**˂0.001**
Lutein (µg/day)^a^	768.1 (569.4)	1452.4 (940.9)	**˂0.001**	**˂0.001**
Lycopene (µg/day)^a^	3530.3 (6242.2)	12,546.5 (6126.3)	**˂0.001**	**˂0.001**
Zinc (mg/day)^b^	6.1 ± 2.5	9.0 ± 2.7	**˂0.001**	**˂0.001**
Selenium (mg/day)^a^	56.4 (33.1)	67.1 (30.6)	**0.006**	**0.002**

*BMI* body mass index, OBS oxidative balance score, *SFA* saturated fatty acids, *MUFA* monounsaturated fatty acids, *PUFA* polyunsaturated fatty acids, MET metabolic equivalent of task.

Values are median (IQR), mean ± SD, or percentage.

P-value less than 0.05 was considered significant.

^a^Wilcoxon U-test has been used.

^b^Paired sample T-test has been used.

^c^McNemar test has been used.

^*^Adjusted for age by ANCOVA test.

The study population nutrient intakes are shown in Table 3. Nutrient intakes of the total population were compared between different tertiles of OBS. Based on the table, participants in the last tertile of OBS had significantly higher intakes of carbohydrates and lower intakes of SFA, monounsaturated fatty acids (MUFA), and PUFA compared to the first tertile (P˂0.001 for all except SFA).

**Table 3:** Consumption of macronutrient intake based on OBS tertile.

Variables	T1 (n = 56)	T2 (n = 54)	T3 (n = 50)	P-value^a^
Carbohydrate (% energy)	57.11 (10.87)	64.81 (6.39)	67.20 (9.12)	**˂0.001**
Protein (% energy)	13.82 (4.12)	13.86 (2.70)	13.53 (2.45)	0.979
SFA (% energy)	8.65 (4.01)	7.60 (4.03)	7.13 (2.48)	**0.001**
MUFA (% energy)	10.32 (3.58)	8.17 (1.86)	7.42 (2.16)	**˂0.001**
PUFA (% energy)	7.06 (2.90)	5.36 (1.40)	5.10 (1.33)	**˂0.001**

*OBS* oxidative balance score, *T* tertile, *SFA* saturated fatty acids, *MUFA* monounsaturated fatty acids, *PUFA* polyunsaturated fatty acids.

Values are median (IQR).

P-value less than 0.05 was considered significant.

^a^Kruskal–Wallis U-test has been used.

Bivariate and multivariable-adjusted odds ratios (ORs) and 95% confidence intervals (CIs) for OBS with sarcopenia are shown in Table 4. In the bivariate model, we observed lower odds of sarcopenia in the second and last tertile of OBS in comparison to the first tertile (T) (T_2_—OR = 0.414, 95% CI: 0.186–0.918 and T_3_—OR = 0.101, 95% CI: 0.041–0.248). After adjusting for potential confounders, the association was not significant in second and last tertile of OBS in comparision to the first one.

**Table 4:** Association between oxidative balance score and sarcopenia.

Variables	Bivariate			Multivariable		
	OR	95% CI	P-value	OR	95% CI	P-value
Oxidative balance score						
T_1_ (≤ 15)	Ref.	Ref.	Ref.	Ref.	Ref.	Ref.
T_2_ (16–21)	**0.414**	**0.186–0.918**	**0.030**	0.885	0.322–2.482	0.813
T_3_ (≥ 22)	**0.101**	**0.041–0.248**	**˂0.001**	0.387	0.099–1.514	0.173
Age (year)	**1.155**	**1.066–1.252**	**0.001**	**1.201**	**1.086–1.329**	**˂0.001**
Energy intake (kcal/day)	**0.997**	**0.996–0.998**	**˂0.001**	0.999	0.998–1.001	0.979
Protein intake (g/day)	**0.928**	**0.905–0.952**	**˂0.001**	**0.934**	**0.889–0.981**	**0.007**
Income (Rials), %						
Less than 3 million	Ref	Ref	Ref	Ref	Ref	Ref
3–6 million	1.185	0.592–2.369	0.631	–	–	–
More than 6 million	0.492	0.203–1.191	0.116	–	–	–
Smoking, %						
Yes	Ref.	Ref.	Ref.	Ref.	Ref.	Ref.
No	1.257	0.538–2.708	0.558	–	–	–

Obtained from conditional logistic regression.

*OR* odds ratio, *CI* confidence interval.

Adjusted for variables with p-value < 0.25 in multivariable analysis (Age, energy, and protein intake were included in the multivariable model).

These values are odds ratio (95% CIs).

Significant values are shown in bold.

## Discussion

The present study investigated the association between OBS and sarcopenia in older adults. There was no significant association between OBS and the odds of sarcopenia after adjusted for potential confounders in backward conditional method. This finding is inconsistent with the hypothesis that the redox balance between exposure to anti-oxidants and pro-oxidants is protective against the possibility of sarcopenia.

In the new guidelines, muscle strength is considered one of the main characteristics of sarcopenia to better identify sarcopenia in clinical practice^32^. In fact, sarcopenia, accompanied by the loss of muscle strength and skeletal mass, has adverse clinical consequences^3,33^. In hospitalized patients, this disease is associated with complications such as loss of independence, infection, low quality of life, pressure ulcers, and increased mortality^34^.

The association between OBS and oxidative stress in some diseases, such as some cancers, has already been studied^35,36^. These studies reported a relationship between OBS and oxidative stress in the occurrence of cancers. Diet accounts for 75% of the overall score of OBS^25,37,38^. Therefore, changing the diet, including reducing the consumption of hydrogenated fats, processed meats, and red meat and increasing the consumption of legumes, whole grains, vegetables, fruits, and nuts can help to increase OBS and increase the anti-oxidant status of the body^15^. Therefore, increasing the consumption of the mentioned foods and reducing some others may prevent diseases related to the body's oxidative imbalance. However, the current study could not find an association between sarcopenia and OBS. The reason for the difference in the results of our study with other studies can be due to the different etiology of cancer and sarcopenia and the different way of scoring in the calculation of OBS.

Many factors are involved in the pathogenesis of sarcopenia, including inflammation, malnutrition, inactivity, endocrine changes, and oxidative stress, many of which do not act in isolation^39^. Although it is believed that oxidative stress plays an important role in many diseases, there is no conclusive evidence regarding the association between anti-oxidants and pro-oxidants with particular health outcomes^40^. This discrepancy in the lack of relationship between these diseases and oxidative stress can be caused by inadequate methods of assessing oxidative stress in humans^11^. In epidemiological studies, OBS is a relatively simple tool to investigate oxidative status and its relationship with diseases^41^.

The OBS undoubtedly needs to be modified and discussed further. One of the limitations of the scoring method in this index is that it considers the same weights for exposure to anti- and pro-oxidants, while they do not have the same effect. For example, α-tocopherol has been shown to have a higher redox potential than vitamin C^42^. Thus, the contribution of vitamin E is different compared to vitamin C^42^. Although OBS is a relatively simple index that evaluates the individual's oxidative balance, more anti-oxidant components can be considered to better investigate the relationship between anti- and pro-oxidants^41^.

The study’s results showed an inverse relationship between protein intake and the risk of sarcopenia. A systematic review and meta-analysis study indicated that people with sarcopenia consumed less protein compared to people without sarcopenia^43^. Inadequate dietary protein intake has also been demonstrated to be associated with reduced muscle mass in the elderly due to lower muscle protein synthesis^44^. Furthermore, we found a positive association between age and the chance of sarcopenia. A cross-sectional study also showed that total skeletal muscle mass and total lean body mass decrease linearly with age^45^. In addition, the decrease in muscle function and muscle mass with age has been shown in other studies^46^.

The present study had some limitations. FFQ does not consider the bioavailability of nutrients or may not include all sources of each nutrient, and it may also have recall bias^47^. However, in epidemiological studies, the FFQ is the most commonly used dietary assessment tool and an easy and effective tool for collecting dietary data. Also, OBS considers factors such as lifestyle and diet and does not include the endogenous measures of the cell's anti-oxidant function. In addition, the relatively small sample size and the study's cross-sectional design can be considered limitations of the present research. However, the current study has some strengths. This study is the first to examine the association between OBS and sarcopenia in older adults. Also, considering more dietary anti-oxidants, including beta-cryptoxanthin, lutein, zeaxanthin, lycopene, etc., in the OBS was another strength of this study.

## Conclusions

The present study's findings demonstrated that overcoming exposure to anti-oxidants over pro-oxidants, as illustrated by a higher OBS, is not related to lower odds of sarcopenia in older adults. Examining the relationship between OBS and inflammatory markers in older adults with sarcopenia would be interesting. More studies are needed to confirm the findings.

## Data Availability

Data are available through a reasonable request from the corresponding author.
